# Weak Fault Feature Extraction of Rolling Bearings Based on an Improved Kurtogram

**DOI:** 10.3390/s16091482

**Published:** 2016-09-13

**Authors:** Xianglong Chen, Fuzhou Feng, Bingzhi Zhang

**Affiliations:** 1 Department of Mechanical Engineering, Academy of Armored Forces Engineering, Beijing 100072, China; fengfuzhou@tsinghua.org.cn; 2 Beijing Special Vehicle Research Institute, Beijing 100072, China; zhangbingz@foxmail.com

**Keywords:** correlated kurtosis, redundant second generation wavelet package transform, kurtogram, weak fault diagnosis

## Abstract

Kurtograms have been verified to be an efficient tool in bearing fault detection and diagnosis because of their superiority in extracting transient features. However, the short-time Fourier Transform is insufficient in time-frequency analysis and kurtosis is deficient in detecting cyclic transients. Those factors weaken the performance of the original kurtogram in extracting weak fault features. Correlated Kurtosis (CK) is then designed, as a more effective solution, in detecting cyclic transients. Redundant Second Generation Wavelet Packet Transform (RSGWPT) is deemed to be effective in capturing more detailed local time-frequency description of the signal, and restricting the frequency aliasing components of the analysis results. The authors in this manuscript, combining the CK with the RSGWPT, propose an improved kurtogram to extract weak fault features from bearing vibration signals. The analysis of simulation signals and real application cases demonstrate that the proposed method is relatively more accurate and effective in extracting weak fault features.

## 1. Introduction

Rolling bearings are one of the most common but the most vulnerable parts in mechanical systems. In order to ensure uninterrupted operation and avoid unnecessary losses caused by sudden failure, extraction of weak fault failures of rolling bearings has become a key factor to condition monitoring and fault diagnosis concerning mechanical systems [1,2].

Influenced by heavy background noise and signal transmission paths, fault features of rolling bearings can be greatly compromised. Consequently, it becomes difficult to extract bearing weak fault features, not to mention to diagnose such faults accurately. This issue had been studied by some signal processing methods, such as Wavelet Transform (WT) [3], Empirical Mode Decomposition (EMD) [4], and Local Mean Decomposition (LMD) [5]. By utilizing noise to enhance signal weak features, stochastic resonance (SR) [6] can effectively process signals with low signal-to-noise ratio (SNR). Lei [6] proposed an adaptive stochastic resonance (ASR) method which performs well in extracting weak characteristics for fault diagnosis. By using the sparsity measurements, Tse [7] proposed the sparsogram to determine the resonant frequency bands quickly and detect fault signals with low SNR. Tse [8] further studied the sparsogram with a joint algorithm based on the complex Morlet wavelet filter and genetic algorithm for maximizing the sparsity measurement value. Tse [7,8] verified the superiority of the sparsogram in bearing fault diagnosis. Later, Antoni [9] proposed the infograms, including the SE infogram and the SES infogram, which can capture the signature of repetitive transients in both time and frequency domains. Starting with Bayesian inference, Wang [10] proposed an extended infogram to optimize wavelet filtering for the identification of bearing fault features, and which extract more signatures than the SE infogram.

Antoni [11,12] put forward the method of spectral kurtosis (or SK) for non-stationary feature extraction. Due to its superiority, several in-depth studies have been done in the bearing vibration monitoring and fault diagnosis [13,14,15,16]. Based on short-time Fourier Transform (STFT), Antoni [17] later built a faster computation method for kurtogram to extract transient features in vibration signals. Wang [18] creatively combined the inherent nonlinear structure with kurtogram to extract weak fault features from low SNR bearing vibration signals. However, kurtogram is conditioned by STFT and FIR [19,20], either of which limits its accuracy in extracting transient features from noise signals. To address the above limitations, Lei [20] suggested that wavelet package transform (WPT) be used to construct kurtogram. Wang [21] tentatively experimented the calculation of the envelope power spectrum of WPT nodes to enhance the kurtogram theories. 

With the help of the periodicity feature of impulses, McDonald [22] proposed the correlated kurtosis (CK) to detect cyclic transients more effectively than the kurtosis. Based on redundant lifting scheme, redundant second generation wavelet package transform (RSGWPT) [23] doesn’t depend on Fourier transform. It possesses time invariant properties, so it can not only afford more detailed local time-frequency description of the signals, but also restrict the frequency aliasing components of the analysis. Therefore, it is helpful to identify the bearing fault-sensitive frequency bands and extract weak fault features. Combining CK and RSGWPT, this manuscript proposes an improved kurtogram, which can remedy the drawbacks of STFT and the kurtosis. The effectiveness of this method is demonstrated by simulation and experimental analysis.

The rest of the manuscript includes: correlated theoretical background in Section 2, the methods proposed by the authors in Section 3, the simulation analysis and test applications in Section 4 and Section 5, respectively, and our conclusions in Section 6.

## 2. Theoretical Background

### 2.1. Spectral Kurtosis and Kurtogram

As a complement to the method of power spectral density in indicating transients of signals, spectral kurtosis (SK), which can indicate both the presence and location of transients in frequency domain, was first introduced by Dwyer [24] and further studied by Antoni [11,12,17].

Given the Wold-Cramér decomposition of a non-stationary signal, signal y(n) as the response of a system with time varying impulse response can be expressed as [2,18,21,22]:

(1) $$y(n)=\int_{-1/2}^{+1/2}e^{j2\pi fn}H(n,f)dX(f)$$

where dX(f) is an orthonormal spectral increment and H(n,f) is the complex envelope of y(n) y(n) at frequency f.

The fourth-order spectral cumulant of y(n) is defined as:

(2) $$C_{4y}(f)=S_{4y}(f)-2S_{2y}^{2}(f)$$

where $S_{2y}(f)$ is the time-averaged result of $S_{2y}(n,f)$, $S_{2y}(n,f)$ is an instantaneous moment and measures the energy of the complex envelope.

The SK can be formally defined as:

(3) $$K_{y}(f)=\frac{C_{4y}(f)}{S_{2y}^{2}(f)}=\frac{S_{4y}(f)}{S_{2y}^{2}(f)}-2$$

Considering the presence of added noise addn(n), the SK can be further expressed as:

(4) $$K_{y+addn}(f)=\frac{K_{y}(f)}{{\left[1+\rho (f)\right]}^{2}}$$

where $K_{y+addn}(f)$ is the SK of signal y(n) with added noise addn(n) and ρ(f) is the noise-to-signal ratio.

The SK of a stationary signal is a constant function of frequency and the SK of a stationary Gaussian signal is identically zero. Where there are signal transients, there will be a high spectral kurtosis peak value to be recorded in both time and location, therefore, the SK possesses the dual abilities to detect and localize transients from a signal.

**Figure 1:**
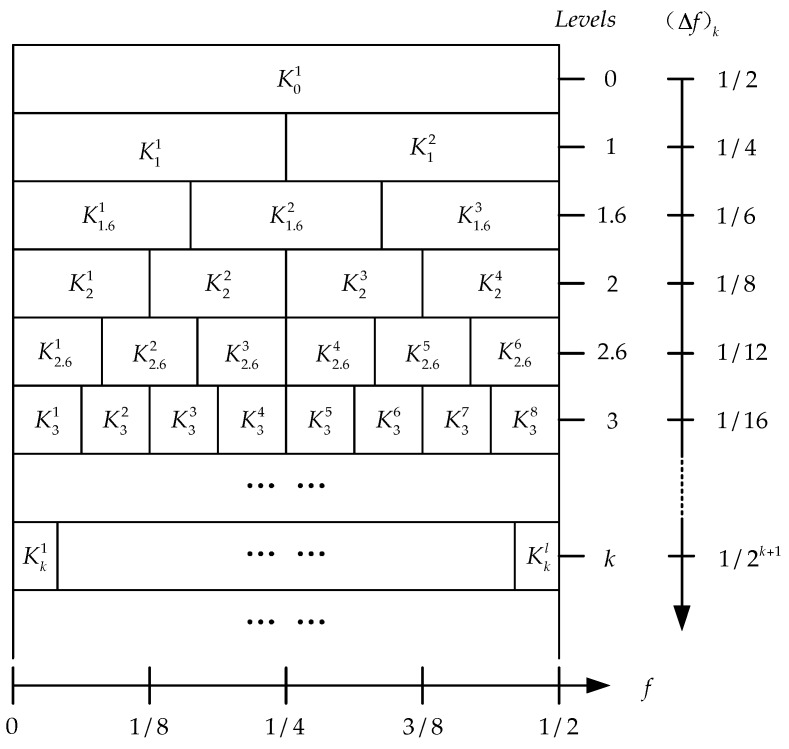
Paving of the original kurtogram proposed by Antoni [20].

In order to improve the calculation efficiency of spectral kurtosis, Antoni [18] utilized binary tree and 1/3-binary tree algorithms to split frequency bands. Based on these calculations, the implementation constructs the kurtogram which is a 2D map and presents values of SK calculated for various frequency band parameters of depth k and bandwidth $(\Delta f{)}_{k}$. Antoni [20] proposed the original kurtogram paving as shown in Figure 1.

### 2.2. Correlated Kurtosis

Spectral kurtosis has been widely applied in rotating machine faults diagnosis for its superiority in detecting transients of no-stationary signals. However, McDonald [22] found that when compared with a signal containing consecutive periodicity of impulses, a signal with a single impulse will generate a higher kurtosis value. It can be concluded that the kurtosis is more effective in detecting a single impulse than consecutive periodicity of impulses. Rotating machines’ vibration signals usually contain consecutive periodicity of impulses, which are produced by mechanical faults, rather than a single impulse, which may be incurred by heavy background noise. Consequently, the kurtosis interfered by heavy noise may indicate wrong transients or wrong locations or both of transients [25], the simulation analysis in Section 4 also verifies this possibility. To address the above problem, McDonald [22] proposed the correlated kurtosis (CK) with the help of the periodicity feature of impulses which exist in signals.

The CK of zero-mean signal Y can be formally defined as [22]:

(5) $$CK_{1}(T)=\frac{\sum_{n=1}^{N}{\left(y_{n}\cdot y_{n-T}\right)}^{2}}{{\left(\sum_{n=1}^{N}y_{n}^{2}\right)}^{2}}$$

(6) $$CK_{M}(T)=\frac{\sum_{n=1}^{N}{\left(\prod_{m=0}^{M}y_{n-mT}\right)}^{2}}{{\left(\sum_{n=1}^{N}y_{n}^{2}\right)}^{M+1}}$$

where N is the number of samples in the input signal Y, T is the periodicity of impulses, and M is the CK shift. Equation (5) is the first shift CK and Equation (6) is the *M*-th shift CK. Equation (6) is more general than Equation (5). And especially when T=0 and M=1, Equation (6) is equal to the norm of kurtosis. Higher shift CK can be used to test a larger series of impulses in a signal. M can be selected based on different fault signals.

McDonald [22] verified that CK approached a maximum about the specified period as opposed to the kurtosis which tended to a maximum with a single impulse. CK takes advantage of the periodical feature of the faults as well as the impulse-like vibration behavior associated with most types of faults. It also can decrease the inference of heavy noise and is more effective in detecting signal cyclic transients.

### 2.3. Redundant Second Generation Wavelet Package Transform

Second generation wavelet transform (SGWT) is a new wavelet construction method using lifting scheme in time domain. It does not depend on Fourier transform and it can construct bi-orthogonal wavelet basis function flexibly. By designing prediction operator and update operator, it can adaptively achieve no-linear wavelet transform [3]. Compared with traditional wavelet transform, it possesses time invariant property and, therefore, can not only afford more detailed local time-frequency description of the signal, but also restrict the frequency aliasing components of the analysis owing to the negligence of the split and merge steps in the decomposition and reconstruction stage [3]. Zhou [23] proposed redundant second generation wavelet package transform (RSGWPT). The construct process is shown as follows.

(1) The prediction step and update step of RSGWPT at level l are performed by $P^{l}$ and $U^{l}$, which are expressed as follows:
  (7) $$\left\{ \begin{array}{l} X_{l+1,2}=X_{l,1}-P^{l+1}(X_{l,1})\\ X_{l+1,1}=X_{l,1}+U^{l+1}(X_{l+1,2})\\ \;\ldots \\ X_{l+1,2^{l+1}}=X_{l,2^{l}}-P^{l+1}(X_{l,2^{l}})\\ X_{l+1,2^{l+1}-1}=X_{l,2^{l}}+U^{l+1}(X_{l+1,2^{l+1}}) \end{array}\right.$$
  where $X_{l,k}$ is the *k-*th wavelet node coefficients at level l, $P^{l}$ is the redundant prediction operator at level l and $U^{l}$ is the redundant update operator at level l.
(2) The reconstruction stage of RSGWPT can be obtained from its decomposition stage, which is expressed as follows:
  (8) $$\left\{ \begin{array}{l} X_{l,2^{l}}=\frac{1}{2}(X_{l+1,2^{l+1}-1}-U^{l+1}(X_{l+1,2^{l+1}})+X_{l+1,2^{l+1}}+P^{l+1}(X_{l+1,2^{l+1}-1}-U^{l+1}(X_{l+1,2^{l+1}})))\\ \;\ldots \\ X_{l,1}=\frac{1}{2}(X_{l+1,1}-U^{l+1}(X_{l+1,2})+X_{l+1,2}+P^{l+1}(X_{l+1,1}-U^{l+1}(X_{l+1,2}))) \end{array}\right.$$
(3) The redundant prediction operator $P^{l}$ and the redundant update operator $U^{l}$ at level l are expressed as follows:
  (9) $$P_{j}^{l}=\left\{ \begin{array}{l} p_{m},\;j=2^{l}m\\ 0,\;j\neq 2^{l}m \end{array}\right.\;(j=1,2,\ldots ,2^{l}N)$$
  (10) $$U_{j}^{l}=\left\{ \begin{array}{l} u_{n},\;j=2^{l}n\\ 0,\;j\neq 2^{l}n \end{array}\right.\;(j=1,2,\ldots ,2^{l}\tilde{N})$$
  where $P=\{p_{m}\}$, m=1,2,⋯,N and $U=\{u_{n}\}$, $n=1,2,\cdots ,\tilde{N}$ are initial prediction operator and initial update operator of SGWT, N and $\tilde{N}$ are their length.

**Figure 2:**
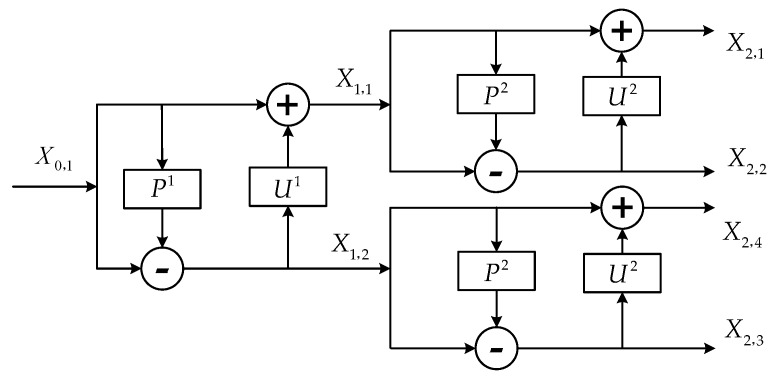
An example of two levels RSGWPT decomposition stage.

**Figure 3:**
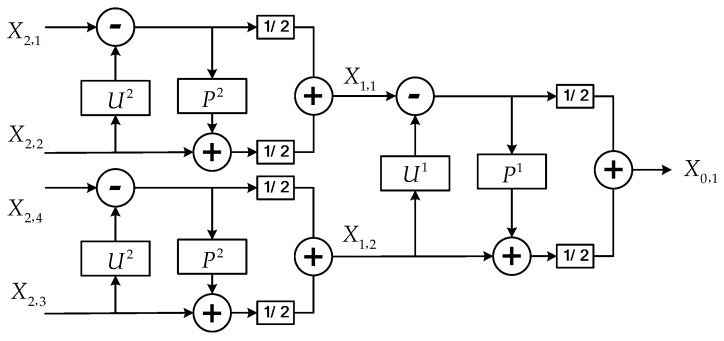
An example of two levels RSGWPT reconstruction stage.

An example of two levels RSGWPT decomposition stage and reconstruction stage are shown in Figure 2 and Figure 3.

Based on redundant lifting scheme, RSGWPT possesses time invariant property which can acquire richer feature information and more precise frequency localization information [23,26]. It can restrict the frequency aliasing components of the analysis. Therefore, RSGWPT is superior to WPT and STFT in time-frequency analysis, and it is helpful to identify fault-sensitive frequency bands which can be used to extract weak fault features. 

## 3. Proposed Method

Under the influence of factors such as heavy background noise and signal transmission paths, fault-sensitive frequency bands will be easily masked. It causes serious challenges on the detection and diagnosis of bearing faults. The original kurtogram put forward by Antoni [17] can be adaptive in identifying fault features when the bearing failure occurs. Therefore, it becomes a useful tool to detect and diagnose bearings’ fault. Yet, with regard to capabilities in time-frequency analysis, the original kurtogram, employing either STFT or FIR, is comparatively inferior to WPT. Based on WPT, Lei [20] and Wang [21] put forward an improved kurtogram and an enhanced kurtogram. However, WPT also has innate limitations such as frequency aliasing, fault features unidentifiable and bearing failures difficult to detect. Nevertheless, the original kurtogram is more effective to a single impulse and more vulnerable to the interference of noise signal [26]. It could not precisely and accurately detect transients or their positions in the frequency domain. All of these reasons restrain kurtogram application in the detection of bearings’ failures under the circumstances of heavy noise.

**Figure 4:**
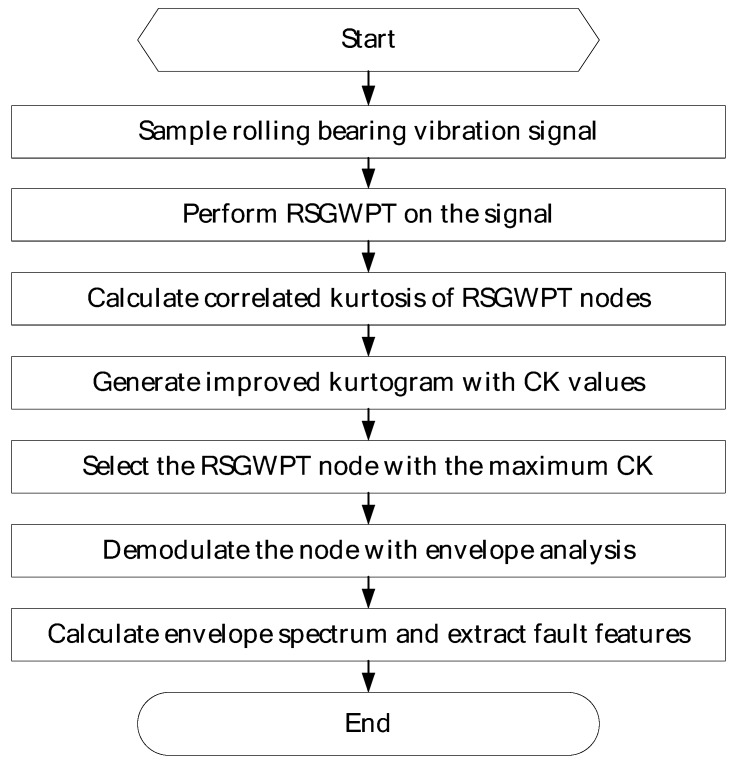
Flow chart of the proposed method.

As is mentioned above, CK approaches a maximum for consecutive impulses about the specified period as opposed to the kurtosis which tends to a maximum with just single impulse. It can also reduce the interference of noise in detecting impulse series. RSGWPT possesses time invariant property. It can restrict the frequency aliasing components of the analysis. It also can acquire richer feature information and more precise frequency localization information. As a result, RSGWPT is superior to WPT and STFT in time-frequency analysis, and it is helpful to identify fault-sensitive frequency bands, which can facilitate the extraction of weak fault features. Owing to the superiority of CK and RSGWPT, by replacing kurtosis with correlation kurtosis and STFT with RSGWPT, this manuscript proposes an improved kurtogram, which can steadily identify fault-sensitive frequency bands and efficiently extract weak fault features. The proposed method, coupled with the envelop analysis, can be employed to extract bearings’ weak fault features and diagnose bearings’ faults, as is shown in Figure 4.

## 4. Simulation Analysis

Rolling bearing vibration signals usually contain consecutive periodicity of impulses, when failure occurs at inner ring, the outer ring, rolling element and the cage. In order to verify the proposed method, a rolling bearing vibration model referred to [27,28] is used to construct bearing failure simulation signals.

Rolling bearing vibration model is expressed as follows:

(11) $$\left\{ \begin{array}{l} x(t)=s(t)+n(t)=\sum_{i}A_{i}h(t-iT-\tau_{i})+n(t)\\ h(t)=\exp(-Ct)\cos(2\pi f_{n}t)\\ A_{i}=A_{0}\cos(2\pi f_{r}t) \end{array}\right.$$

where T is the average period of impulse series. $f_{i}$ is the fault feature frequency which equals to the reciprocal of T and is set to 100 Hz, $f_{r}$ is the rotating frequency which equals to 20 Hz, $f_{n}$ is the fault-sensitive resonance frequency which equals to 4000 Hz, $f_{s}$ is the sampling frequency which is set to 12,800 Hz, $\tau_{i}$ is the tiny random fluctuation of the *i*-th impulse and $\tau \sim N(0,0.05/f_{r})$, C is the damping coefficient which equals to 900, and the sampling points are set to 12,800. 

**Figure 5:**
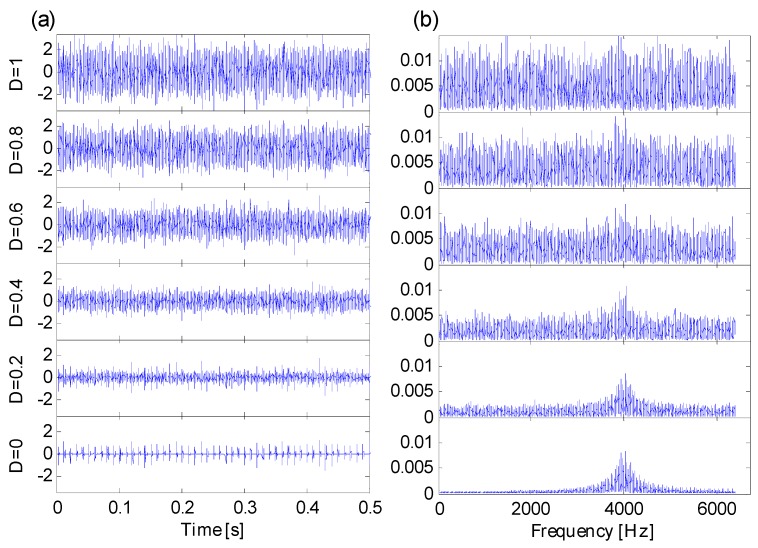
(**a**) Six simulation signals in time domain; (**b**) six simulation signals in frequency domain.

In line with Equation (11), simulation signals with Gaussian noise are generated as is shown in Figure 5. When noise variation D≥0.6, both the impulse series and the fault-sensitive resonance frequency band are totally masked by heavy background noise, there are no certain resonance frequency bands that can be directly detected from frequency spectrums. Heavy noise leads to difficulties in extracting fault features from raw signals.

In order to verify the effectiveness of the proposed method in identifying fault-sensitive resonance frequency bands and extracting weak fault features with heavy background noise, a simulation signal with noise variation D=0.8 is designated to test the proposed methods. The simulation signal’s transient features are overwhelmed by heavy background noise and its fault-sensitive resonance frequency band is obscure, so the simulation signal is appropriate to verify the effectiveness of the proposed method.

**Figure 6:**
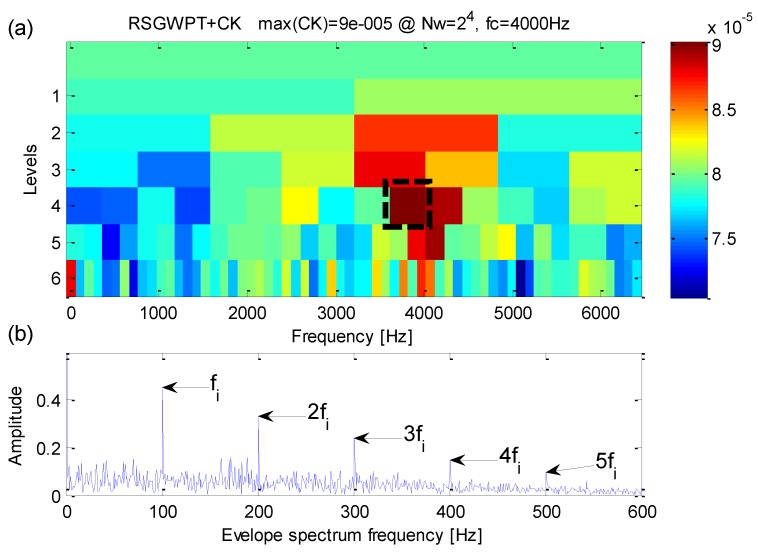
(**a**) The results of the improved kurtogram; (**b**) the results of the corresponding envelope spectrum.

Analysis of the simulation signal performed by the proposed method is shown in Figure 6. From Figure 6a, one can find that the maximum correlated kurtosis occurs at level 4 with a frequency bandwidth *f_RCK_bw_* = 400 Hz and a band central frequency *f_RCK_c_* = 4000 Hz which is inconsistent with the fault-sensitive resonance frequency of the simulation signal. From Figure 6b, the fault feature frequency *f_i_* = 100 Hz and its harmonic frequencies are easy to identify. In the meanwhile, fault feature frequencies are dominant in frequency domain.

**Figure 7:**
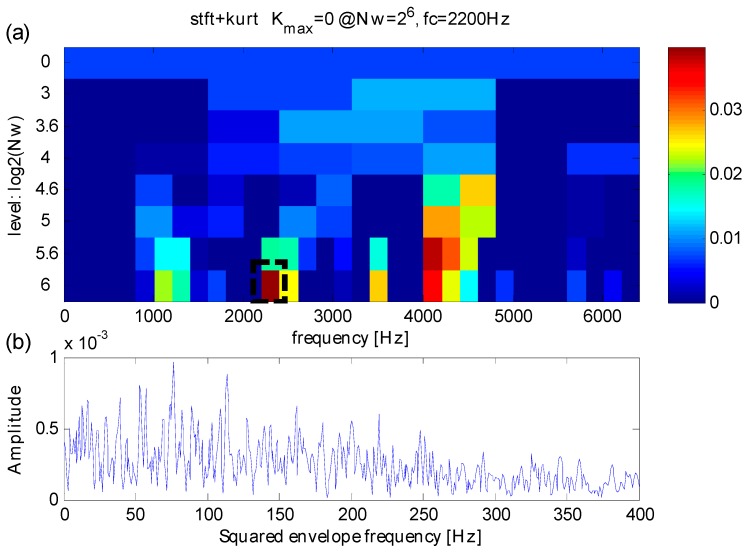
(**a**) The results of original kurtogram; (**b**) the reuslts of squared envelope spectrum.

Analysis of the same simulation signal obtained by the original kurtogram is shown in Figure 7. With the interference of heavy noise, one can find from Figure 7a,b that the maximum kurtosis occurs at level 6 with a band central frequency *f_FK_c_* = 2200 Hz. The frequency band extracted via maximum kurtosis is different from the fault-sensitive resonance frequency *f_n_* = 4000 Hz. The width of the extracted frequency band is *f_FK_bw_* = 100 Hz. By contrast, the original kurtogram indicates the fault-sensitive resonance frequency improperly and the results hence obtained incorrect.

**Figure 8:**
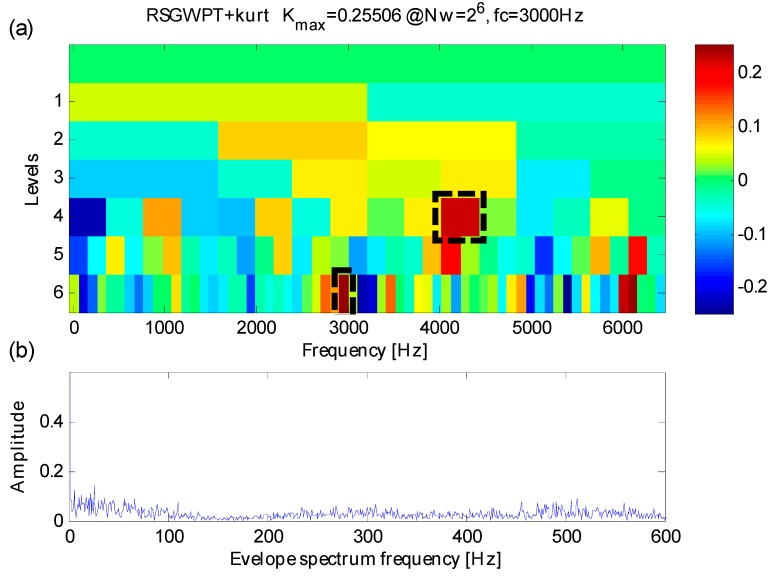
(**a**) The results of kurtogram; (**b**) trhe results of envelope spectrum.

In order to illustrate the necessity and effect of RSGWPT used in the proposed method, a kurtogram is constructed only by RSGWPT and the kurtosis, which is different from the original kurtogram. The analysis is shown in Figure 8. The maximum kurtosis node occurs at level 6 of RSGWPT with a central frequency *f_RK_c_* = 3000 Hz and a bandwidth *f_RK_bw_* = 100 Hz, and its corresponding envelope spectrum doesn’t contain any noticeable fault feature frequencies. However, one can learn from Figure 8a that the constructed kurtogram contains an identifiable kurtosis peak value at level 4 around the fault-sensitive resonance frequency *f_n_* = 4000 Hz. The RSGWPT is usually believed to be superior to either WPT or STFT in time-frequency analysis but not to such a degree that the effect of kurtosis in detecting transient components might be free from interference of heavy noise, making the RSGWPT fail to identify appropriate fault-sensitive frequency band or to demodulate the frequencies fault features. 

To further the previous studies, the proposed method, by combining RSGWPT and correlated kurtosis, can facilitate the detection of transients and the extraction of weak fault features, just as is shown in Figure 6. Thus, the necessity of the combination between RSGWPT and CK in the proposed method has been proved, and the effect of the proposed method in extracting weak fault features of signals with heavy background noise has been verified in turn.

**Figure 9:**
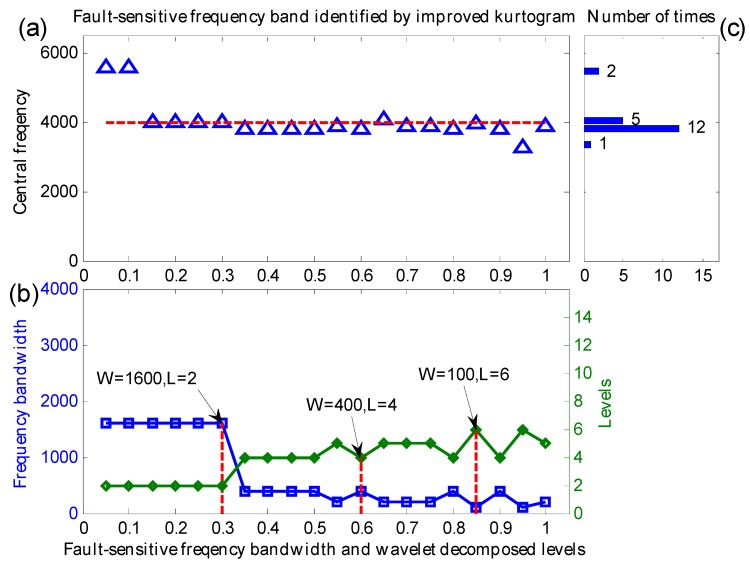
(**a**) The central frequencies; (**b**) the frequency bandwidth and decomposition levels; (**c**) distribution of central frequencies.

**Figure 10:**
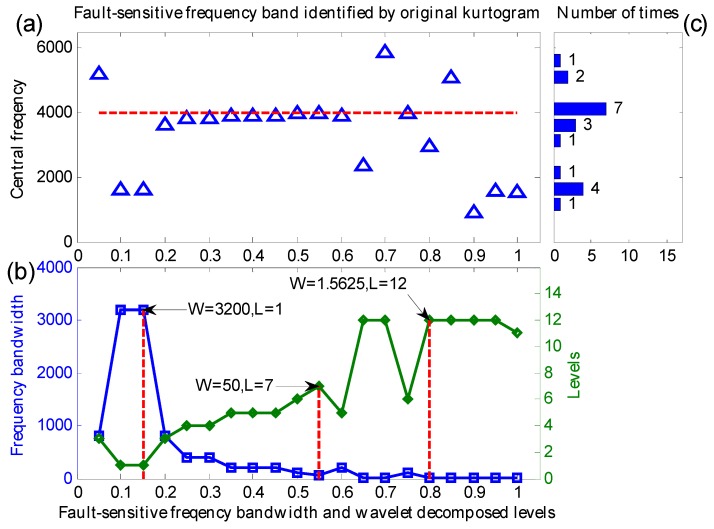
(**a**) The central frequencies; (**b**) the frequency bandwidth and decomposition levels; (**c**) distribution of central frequencies.

Similarly, the manuscript selects a set of 20 simulation signals generated by Equation (11) with a Gaussian noise mean of 0 and different noise variation (varying from 0.05 to 1 with step length 0.05) to verify the effect of the proposed method in extracting weak fault features. Both the proposed method and the original kurtogram are utilized to analyze these signals, their central frequencies, frequency bandwidth and decomposition levels of the identified fault-sensitive frequency bands are shown in Figure 9 and Figure 10, respectively.

One can learn that, from Figure 9a, within the range of the noise variation between 0.15 and 1, the indicated maximum correlated kurtosis frequency bands are adjacent to the real fault-sensitive frequency *f_n_* = 4000 Hz. From Figure 9b, the indicated maximum correlated kurtosis frequency bands with adequate bandwidth fall between appropriate range scales. From Figure 9c, the indicated central frequencies of maximum correlated kurtosis frequency bands are more concentrated, neighboring on the real fault-sensitive frequency. Consequently, the proposed method can steadily identify the fault sensitive resonance frequency band, which is helpful to demodulate fault features exactly.

From Figure 10a, the maximum kurtosis frequency bands indicated by the original kurtogram are also close to the real fault sensitive frequency in the noise variation range between 0.2 and 0.6. Nevertheless, from Figure 10b, the indicated maximum kurtosis frequency bands have a larger fluctuation range of decomposition levels and narrow bandwidths. From Figure 10c, the indicated maximum kurtosis frequency bands have deviated central frequencies and it is useless to extract fault features. Moreover, within the noise variation range from 0.65 to 1, the original kurtogram is interpreted by heavy noise and indicates an incorrect location of the transients in frequency domain. Consequently, their envelope spectrums are hard to extract correct weak fault features.

As mentioned above, it cannot identify fault-sensitive frequency bands effectively or extract weak fault features correctly because the original kurtogram is restricted by limitations of STFT and kurtosis in detecting consectutive impulses. However, the proposed method based on RSGWPT and CK can be steadier in identifying fault-sensitive frequency bands. Accordingly, it can correctly extract weak fault features of simulation signals with heavy background noise.

## 5. Applications

Data sets from the Case Western Reserve University (CWRU) Bearing Data Center and vibration signals from a rolling bearing of a real transmission are utilized to verify the validity of the proposed method.

### 5.1. Case 1: Extraction Test Based on Bearing Data from CWRU

**Figure 11:**
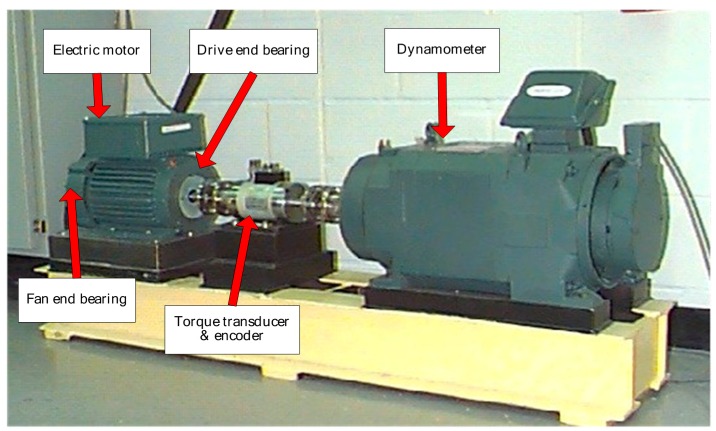
The bearing test rig of the CWRU Bearing Data Center [29].

The bearing test rig of the CWRU Bearing Data Center [29] is shown in Figure 11, which consists of a two horsepower Reliance Electric motor, a torque transducer/encoder, a dynamometer and control electronics. Motor bearings are seeded with faults using electro-discharge machining. Faults ranging from 0.007 inches to 0.040 inches in diameter are introduced separately on the inner ring, rolling element and outer ring. Faulty bearings are then reinstalled into the test rig and motor loads ranging from 0 to 3 horsepower and motor speeds rotating at from 1797 to 1720 RPM are applied. Vibration data are collected using accelerometers, which are mounted at the 12 o’clock position at both the drive end and fan end of the motor housing. Vibration data sets are recorded using a data acquisition system and the sampling frequency is set to 12 KHz for some experiments and 48 KHz for others. The details of faulted bearing and frequencies of faults are shown in Table 1. Further details regarding the test setup can be found on the CWRU Bearing Data Center Website [1,29].

Data sets from CWRU Bearing Data Center have been a standard reference used to test diagnosis algorithms in the field of bearing diagnosis. In order to examine the CWRU data sets thoroughly and to classify them appropriately, Smith [1] firstly achieved a benchmark study by using three established benchmark fault diagnostic methods which respectively are envelope analysis of the raw signal (Method 1), cepstrum pre-whitening (Method 2) and the original kurtogram (Method 3). With classifications and recommendations given by Smith, both data X171_FE and X224_DE cannot diagnose clearly by three established benchmark methods. So, data X171_FE and data X224_DE are used to verify the validity and superiority of the proposed method.

**Figure 12:**
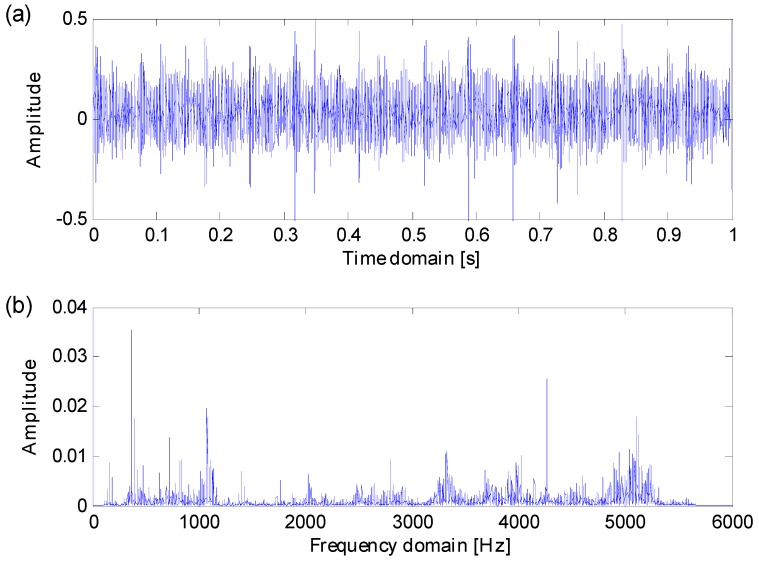
(**a**) Data X171_FE in time domain; (**b**) the spectrum of X171_FE in frequency domain.

**Figure 13:**
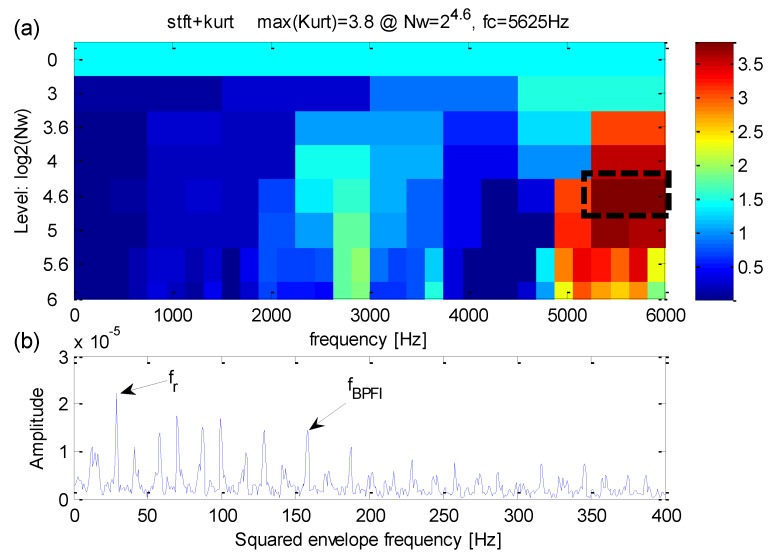
(**a**) The kurtogram of data X171_FE; (**b**) the squared envelope spectrum of the frequency band with the maximum kurtosis.

**Figure 14:**
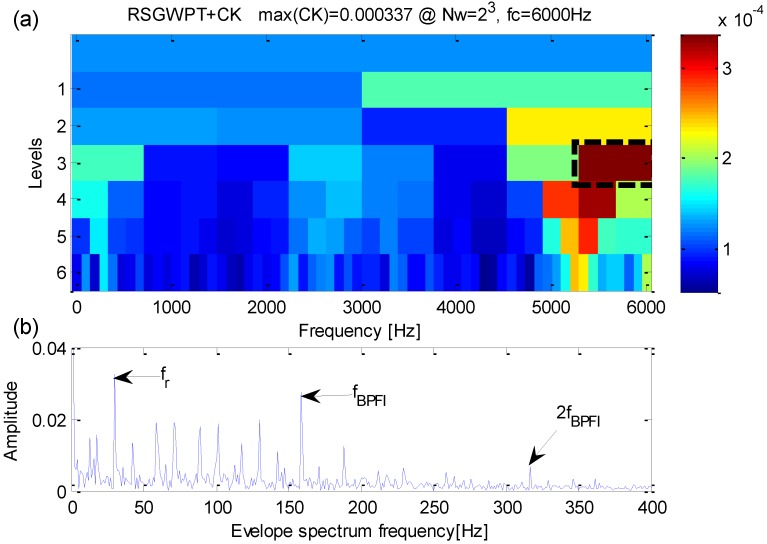
(**a**) The improved kurtogram of data X171_FE; (**b**) the envelope spectrum of the frequency band with the maximum correlated kurtosis.

Data X171_FE (inner ring faults, sampling frequency is set to 12 KHz, rotating speed equals to 1752 RPM, inner ring fault frequency equals to 158.1 Hz) are used as benchmark data to compare between the proposed and the original kurtogram method. X171_FE and its frequency spectrum are shown in Figure 12. The results obtained by the original kurtogram are shown in Figure 13. One can learn from Figure 13b that the squared envelope spectrum not only exhibits obvious rotating frequency and its harmonics, but also indicates fault frequency *f_BFPI_*. However, just as the benchmark study of Smith found that the squared envelope spectrum shows discrete components at the expected fault frequencies but they are not dominant in the spectrum. The results obtained by the proposed method are shown in Figure 14. One can learn from Figure 14b that the fault frequency and its harmonics of the envelope spectrum are more obvious than those in Figure 13b and that the fault frequency has a much higher value than its side frequencies while it is not true with Figure 13b. However, influenced by the obvious rotating frequency, its harmonics and side frequencies, the fault frequency and its harmonics in Figure 14b are not extraordinarily dominant in the spectrum, but it really obtains enhanced results than those in Figure 13b.

**Figure 15:**
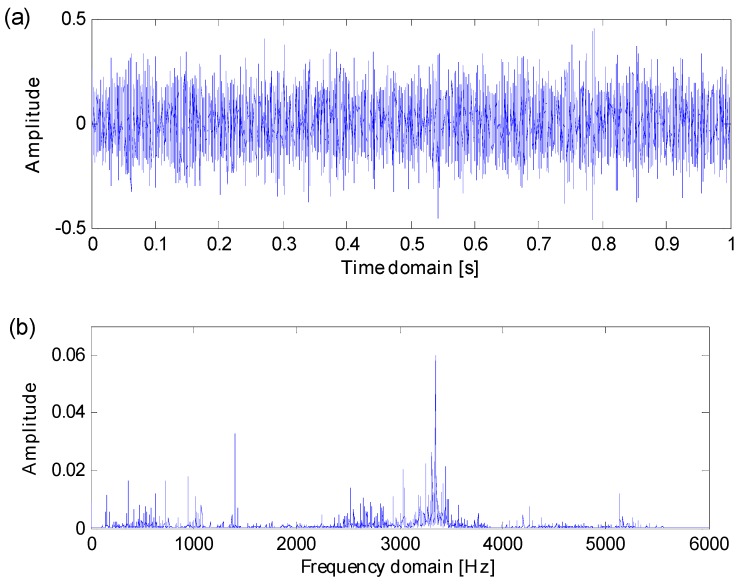
(**a**) Data X224_DE in time domain; (**b**) the spectrum of X224_DE in frequency domain.

**Figure 16:**
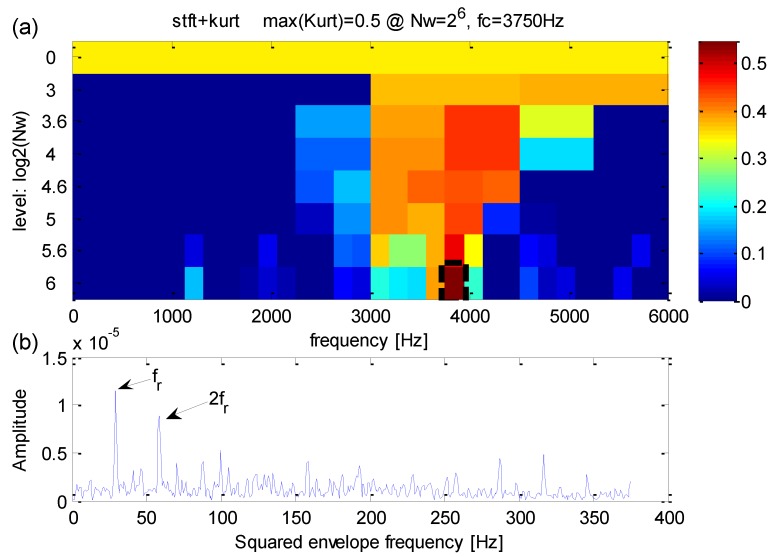
(**a**) The kurtogram of data X224_DE; (**b**) the squared envelope spectrum of the frequency band with the maximum kurtosis.

**Figure 17:**
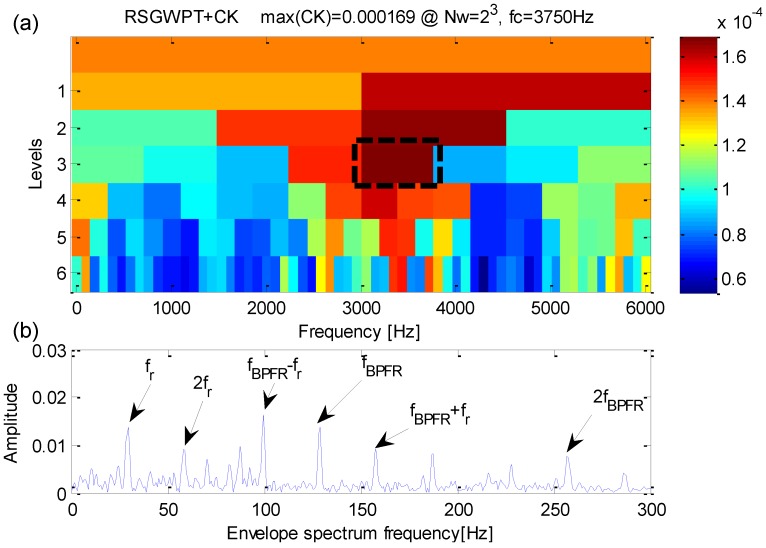
(**a**) The improved kurtogram of data X224_DE; (**b**) the envelope spectrum of the frequency band with the maximum correlated kurtosis.

Data X224_DE (rolling element faults, sampling frequency is set to 12 KHz, rotating speed equals 1754 RPM, rolling element fault frequency equals 137.8 Hz) are also used as benchmark data to compare between the proposed and the original kurtogram method. X224_DE and its frequency spectrum are shown in Figure 15. As the benchmark study of Smith mentions, data X224_DE are not diagnosable for the specified bearing fault by three established methods, including the original kurtogram method. The results obtained by the original kurtogram are shown in Figure 16. One can learn from Figure 16b that, with the influences of STFT and heavy noise, the maximum kurtosis frequency band occurs at level 6, the obtained squared envelope spectrum doesn’t contain any clearly fault frequencies except rotating frequency and its harmonics. The results obtained by the proposed method are shown in Figure 17. One can learn from Figure 17b that the envelope spectrum exhibits clearly fault frequency, its harmonics and side frequencies. 

As is mentioned above, the proposed method can effectively highlight fault frequencies and extract correct bearing fault features. CWRU data has verified the validity and superiority of the proposed method.

### 5.2. Case 2: Extraction Test Based on Data from a Real Transmission Rolling Bearing

**Figure 18:**
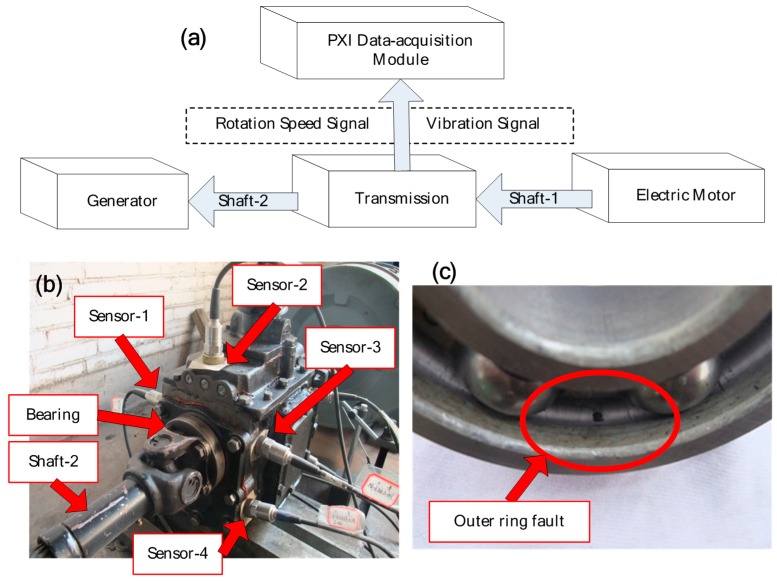
(**a**) Structure of the experiment system; (**b**) locations of sensors, bearing and shaft; (**c**) the faulted bearing.

A real transmission experiment system is shown in Figure 18, which consists of an automobile transmission of BJ2020S, an electric motor, a generator and a data-acquisition module, as exactly shown in Figure 18a. The electric motor is used to drive the transmission and the generator to simulate system load. Vibration data are collected by using accelerometers, which are mounted at locations neighboring on the faulted bearing. Locations are shown in Figure 18b. The bearing of the output shaft (Shaft-2) is seeded with an outer ring fault using electro-discharge machining, as exactly shown in Figure 18c. Vibration data sets are recorded using a PXI data acquisition module and the sample frequency is set to 40 KHz. The faulted bearing details and outer ring fault frequencies are shown in Table 2.

**Figure 19:**
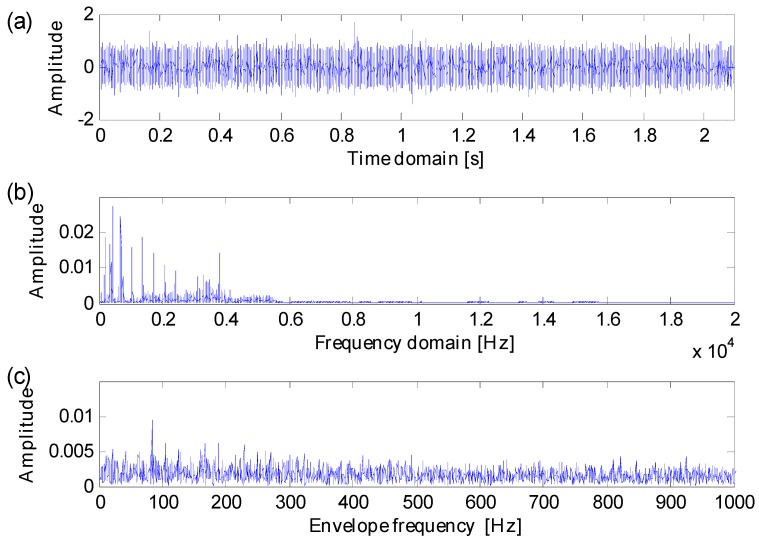
(**a**) Vibration signal; (**b**) frequency spectrum; (**c**) envelope spectrum.

Figure 19 displays a recorded vibration signal, the corresponding frequency spectrum and envelope spectrum. With the influence of heavy noise, one cannot find any impulse components from the vibration signal. And the fault feature frequencies are completely masked by heavy noise in the envelope spectrum.

**Figure 20:**
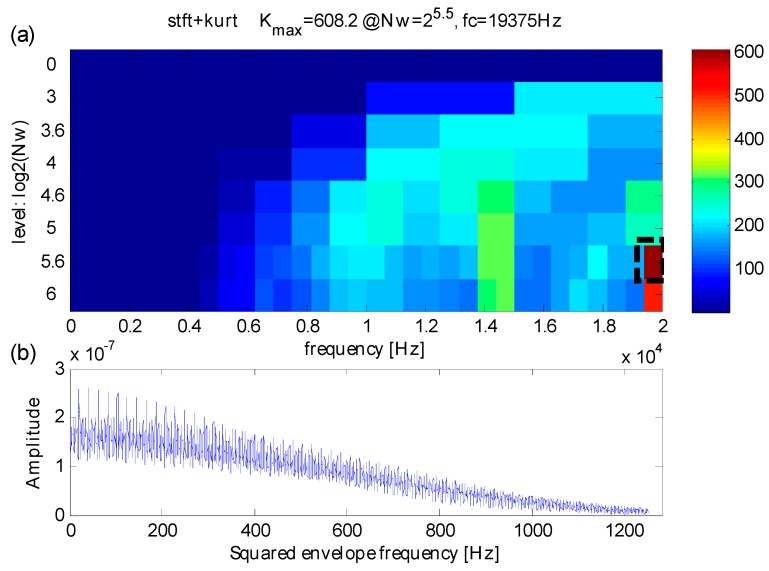
(**a**) The kurtogram; (**b**) the squared envelope spectrum of the frequency band with the maximum kurtosis.

Firstly, the original kurtogram is used to extract fault features by analyzing the signal which is shown in Figure 19a, the results are shown in Figure 20. According to the kurtogram presented in Figure 10a, the maximum kurtosis is calculated at the 5.6th decomposition level and the corresponding frequency band has a central frequency *f_FK_c_* = 19375 Hz and a bandwidth *f_FK_bw_* = 625 Hz. The squared envelope spectrum of the frequency band with maximum kurtosis is presented in Figure 20b, which has a large scope of masked frequencies, therefore it becomes more difficult to identify correct weak fault frequencies.

**Figure 21:**
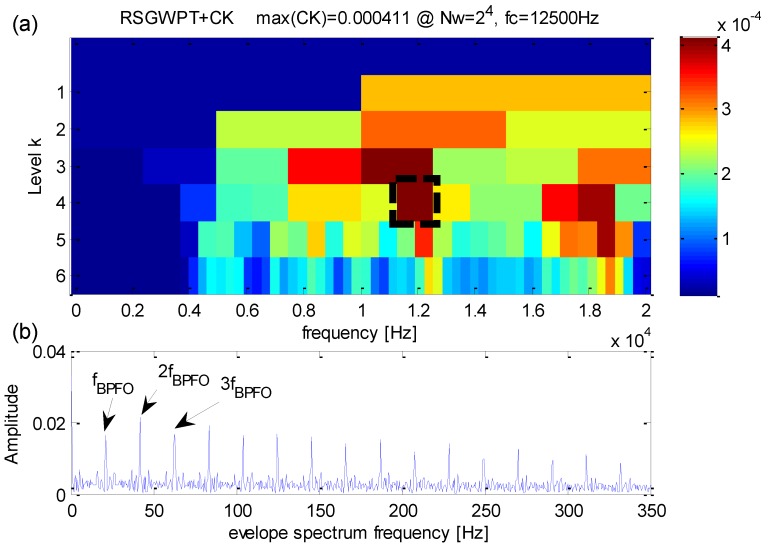
(**a**) The improved kurtogram; (**b**) the envelope spectrum of the frequency band with the maximum correlated kurtosis.

The same as the CWRU case, the effectiveness of the proposed method is to be demonstrated in actual test of fault features extraction. The results of the same signal are shown in Figure 21. According to the proposed kurtogram presented in Figure 21a, the maximum correlated kurtosis is calculated at the 4th RSGWPT decomposition level and its corresponding frequency band has a central frequency *f_RCK_c_* = 12500 Hz and bandwidth *f_RCK_bw_* = 1250 Hz. It can be observed that the fault frequency and its harmonics are quite efficiently extracted. At the same time, they are dominant in the frequency domain. Therefore, it may be concluded that, by comparison with the original kurtogram, the proposed method can successfully extract fault features from signals despite the heavy background noise.

**Figure 22:**
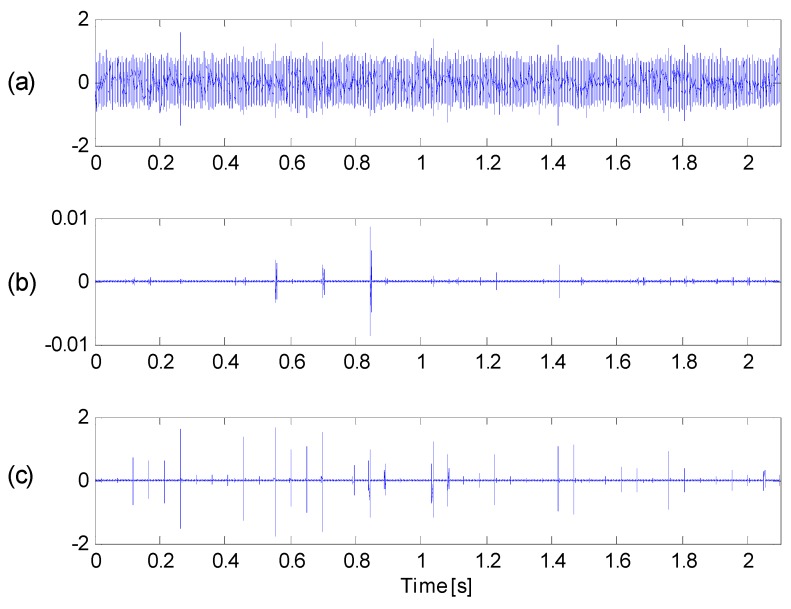
(**a**) The original signal; (**b**) signal filtered by the original kurtogram; (**c**) signal filtered by the proposed method.

In order to further demonstrate the effectiveness of the proposed method, the original signal, the filtered signal with maximum kurtosis via original kurtogram and the filtered signal with maximum correlated kurtosis via the proposed method are separately presented in Figure 22a–c. 

With influence of heavy background noise, one cannot find any impulses from Figure 22a. Several impulses can be found in Figure 22b, but there is no sign of periodicity. Besides, the weak impulses are not clear enough, either. However, in Figure 22c, the filtered signal via the proposed method has clearly exhibit consecutive periodicity of impulses and there is significant sign of periodicity which coincides with the fault feature frequency. Thus, it can be concluded that the proposed method is more efficient in extracting fault feature despite heavy noise. And this application test with real case verifies the validity and superiority of the proposed method.

## 6. Conclusions

For the inherent limitations of the kurtogram, this manuscript put forward an improved version of kurtogram, which has been proved more effective in the extraction of weak fault features of rolling bearing signals. Regarding the proposed approach, the CK can reduce the noise impact on the detection of impulse consequences and approach a maximum for a periodic impulse about the specified period as opposed to the kurtosis which tends to a maximum with a single impulse; the RSGWPT, based on redundant lifting scheme and possessing time invariant property, can not only acquire richer feature information and more precise frequency localization information, but also restrict the frequency aliasing components of the analysis results, the RSGWPT is confirmed to be superior to WPT and STFT in time-frequency analysis, and hence helpful to detect fault-sensitive frequency bands which materialize the extraction of weak fault features. Combining the advantages of CK with those of RSGWPT, this manuscript proposes an improved kurtogram. The proposed method, coupled with the envelop analysis, enable the researchers extract bearing weak fault features from signals against heavy background noise. Analysis results of simulation signals, CWRU data and a real transmission bearing vibration signals demonstrate that, the proposed method is effective in extracting weak fault features from rolling bearing vibration signals.

## Figures and Tables

**Table 1:** Bearing details and fault frequencies.

Position on Rig	Bearing Model	Fault Frequencies (Multiple of Running Speed in Hz)			
		Inner Ring	Outer Ring	Cage Train	Rolling Element
Drive end	SKF 6205-2 RS JEM	5.415	3.585	0.398	4.714
Fan end	SKF 6203-2 RS JEM	4.947	3.053	0.382	3.987

**Table 2:** The faulted bearing details and outer ring fault frequency.

Parameter	Value
Rolling model	6307N
Pitch ring diameter (mm)	57.5
Roller diameter (mm)	14.22
Roller number	7
Contact angel (deg)	0
Rotating speed (rpm)	471
BPFO (Hz)	20.7
